# *QuickStats:* Percentage* of Adults Aged ≥50 Years Who Ever Received a Shingles Vaccination,^†^ by Race and Hispanic Origin**^§^** and Sex — National Health Interview Survey, United States, 2019**^¶^**

**DOI:** 10.15585/mmwr.mm7024a5

**Published:** 2021-06-18

**Figure:**
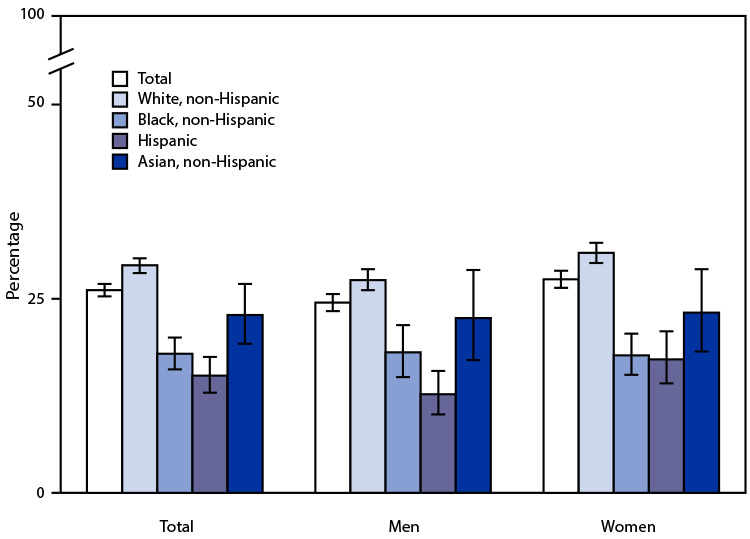
In 2019, 26.1% of adults aged ≥50 years had ever received a shingles vaccination. Non-Hispanic White adults (29.3%) were more likely than non-Hispanic Asian (22.9%), non-Hispanic Black (17.9%), and Hispanic (15.1%) adults to have ever received a shingles vaccination. Overall, women (27.5%) were more likely than men (24.5%) to be vaccinated, and this pattern was consistent for non-Hispanic White women and men (30.9% versus 27.4%) and for Hispanic women and men (17.2% versus 12.7%). No statistically significant difference by sex was observed for non-Hispanic Asian women and men (23.2% versus 22.5%) or non-Hispanic Black women and men (17.7% versus 18.1%).

**Source:** National Center for Health Statistics, National Health Interview Survey, 2019. https://www.cdc.gov/nchs/nhis.htm

